# Deciphering Pathogenesis of Silica Nanoparticle-Induced Airway Remodeling and Fibrosis: Insights from a Human Patient Cohort and a Murine Model

**DOI:** 10.3390/nano16140866

**Published:** 2026-07-15

**Authors:** Aleksandra V. Sen’kova, Innokenty A. Savin, Olga S. Kotova, Ilya S. Shpagin, Elena V. Dmitrienko, Victoriya K. Popova, Bulat R. Khasanov, Alphya R. Tsygankova, Oleg V. Markov, Mona S. Awad, Anatoly I. Saprykin, Lyubov A. Shpagina, Valentin V. Vlassov, Marina A. Zenkova

**Affiliations:** 1 Knorre Institute of Chemical Biology and Fundamental Medicine, Siberian Branch of the Russian Academy of Sciences, Lavrent’ev Ave. 8, 630090 Novosibirsk, Russia; savin_ia@1bio.ru (I.A.S.); elenad@1bio.ru (E.V.D.); fomenkoniboch@gmail.com (V.K.P.); b.khasanov@g.nsu.ru (B.R.K.); markov_ov@1bio.ru (O.V.M.); mona.awad.edu@gmail.com (M.S.A.); vvv@1bio.ru (V.V.V.); 2 Department of Hospital Therapy and Medical Rehabilitation, Novosibirsk State Medical University, Krasny Prospect 52, 630091 Novosibirsk, Russia; ok526@yandex.ru (O.S.K.); mkb-2@yandex.ru (I.S.S.); lashpagina@gbuzgkb2.ru (L.A.S.); 3 Nikolaev Institute of Inorganic Chemistry, Siberian Branch of the Russian Academy of Sciences, Lavrent’ev Ave. 3, 630090 Novosibirsk, Russia; alphiya@yandex.ru (A.R.T.); saprykin@niic.nsc.ru (A.I.S.)

**Keywords:** occupational COPD, nanoparticles, silicon dioxide, clinical cohort, in vivo model

## Abstract

Occupational chronic obstructive pulmonary disease (O-COPD) represents a lung disorder attributable to occupational exposures that are characterized by early development of airway remodeling and pulmonary fibrosis. O-COPD is poorly recapitulated by existing preclinical models. This study aimed to perform comparative characterization of an O-COPD patient cohort exposed to industrial aerosols and to develop a relevant murine model that accurately mirrors the human pathology. In the patient cohort, it was shown that the O-COPD phenotype is associated with the chemical composition of industrial aerosols and mediated by a specific inflammatory pattern with predominant obstructive changes and increased bronchial reactivity upon exposure to metal particles, as well as irreversible fibrotic changes in the lungs upon exposure to silicon dioxide. In the murine model, silica nanoparticles (SiNPs) or magnetic nanoparticles (MNPs) were utilized. Repeated intranasal SiNP administrations have been shown to reflect one of the main features of O-COPD—progressive airway remodeling and fibrosis, observed even after elimination of SiNPs. Administration of MNPs in the same regimen did not result in fibrotic changes in the lungs, partially recapitulating the human pathology resulting from exposure to the complex composition of industrial aerosols as well as the specific properties of chemically synthesized nanoparticles. Thus, the integrative data from the human cohort and animal model provides a reflective platform to advance the investigation of O-COPD mechanisms and development of interventions for fibrotic lung pathology.

## 1. Introduction

Chronic obstructive pulmonary disease (COPD) is one of the leading causes of morbidity and mortality worldwide, representing a significant global health burden [1]. It is a heterogeneous lung disease, characterized by chronic respiratory symptoms (dyspnea, productive cough, and excessive sputum production) resulting from alterations in the airways (bronchitis) and/or alveoli (emphysema) that cause persistent, progressive and irreversible airflow obstruction [2,3]. While cigarette smoking is the primary etiological factor, a substantial proportion of COPD cases are attributable to occupational exposures, classified as occupational COPD (O-COPD), a distinct form of the disease driven primarily by the inhalation of industrial or organic dust, chemicals, gases, or fumes in the workplace. O-COPD can follow a pathophysiological course different from smoking-induced disease, characteristically presenting with a more pronounced fibrotic component [4,5]. These conditions are often diagnosed at late stages, resulting in irreversible lung damage and diminished work capacity [6].

Among multiple occupational hazards, silica exposure is one of the most prevalent in relation to O-COPD [7]. In nature, silicon dioxide (SiO_2_) exists in two forms—crystalline and non-crystalline (amorphous) particles, the latter encompassing silica nanoparticles (SiNPs) that are widely employed in various biological applications [8,9]. Exposure to the crystalline particles in the micrometer size range is well known to induce several respiratory system diseases, including silicosis, COPD and cancer [10]. Today, respirable crystalline silica remains one of the main components of aerosols affecting industrial workers, leading to an increased prevalence of related respiratory diseases [11]. Amorphous silica particles are generally considered less toxic; however, the same cannot be assumed for nanoscale SiNPs, both naturally occurring and chemically synthesized, which are capable of inducing pulmonary inflammation and fibrosis [12]. Due to the growing demand for SiNPs by various industries, such as biomedicine [13] and drug delivery systems [14], occupational exposure to chemically synthesized SiNPs has been steadily rising in recent years [15]. Owing to their small size and high surface area-to-volume ratio, SiNPs penetrate deep into the respiratory system upon inhalation, severely affecting bronchioles and alveoli, while evading clearance mechanisms, and inducing potent oxidative stress and a persistent inflammatory response [16,17]. The resulting structural damage, including the destruction of alveolar walls and the development of irreversible peribronchiolar fibrosis, becomes fixed and self-sustaining, leading to a permanent decline in lung function, with no currently effective treatments [18,19].

The development of effective diagnostic and prognostic models as well as therapeutic approaches for O-COPD relies on robust preclinical models that accurately mirror human pathology. Currently, the most widely used in vivo models, such as chronic cigarette smoke exposure or elastase instillation in rodents, primarily induce inflammation and emphysematous changes [20]. However, these models fail to adequately recapitulate the pronounced airway remodeling and fibrotic characteristics of O-COPD. Thus, there is a critical unmet need for a representative and reproducible animal model that specifically addresses the pathobiological mechanisms triggered by occupational exposures.

Therefore, the aim of our study was to experimentally investigate the development of the primary features of O-COPD in a murine model, evaluate the key morphological signs of disease in the absence of confounding factors (comorbidity, bad habits), and compare them with findings from a human patient cohort. To this end, we utilized a murine model of SiNP-induced pulmonary fibrosis that reflects the specific characteristics of human O-COPD. In parallel, a thorough analysis of the development of O-COPD in industrial workers, as well as its relationship with exposure to occupational hazards of diverse origin, was performed. By integrating these data, our study provides an investigative platform and novel insights to advance the research into the mechanisms and potential interventions for occupational lung diseases.

## 2. Materials and Methods

### 2.1. Patients

A prospective cohort clinical study (from 2019 to 2022) included 50 patients with occupational chronic obstructive pulmonary disease (O-COPD) exposed to multicomponent industrial aerosols during their work time and 50 apparently healthy individuals working under similar conditions. Also, 50 patients with COPD who were tobacco smokers without occupational health risks were recruited to the study. Informed consent was obtained from all patients. The inclusion, non-inclusion, and exclusion criteria are presented in Table S1. During the study, no exclusion criteria were identified for any of the patients. The study also included 50 conditionally healthy volunteers without exposure to industrial aerosols, who constituted the control group.

To determine the phenotype and severity of COPD, all patients underwent the following procedures: assessment of symptom severity using the COPD Assessment Test (CAT) questionnaire; spirometry with a bronchodilator test (MicroLab CareFusion spirometer, CareFusion, Franklin Lakes, NJ, USA) in accordance with the ATS/ERS 2005 standard [21] and Russian Federal Clinical Guidelines with measurement of pre- and post-bronchodilator ratio of forced expiratory volume in 1 s [FEV1] to forced vital capacity [FVC] (FEV1/FVC), FEV1, and inspiratory lung capacity; assessment of bronchial hyperreactivity using an exercise challenge test according to ATS/ERS recommendations (Schiller Intertack 8100T treadmill system, Schiller AG, Baar, Switzerland, R. Bruce protocol); measurement of lung diffusion capacity for carbon monoxide (DLco) by the single-breath breath-holding method (Power Cube Body Diffusion system, GANSHORN Medizin Electronic, Niederlauer, Germany); computed tomography (CT) of the chest (Neusoft NeuViz scanner, Neusoft, Shenyang, China); a six-minute walk test according to the ATS standard; Doppler echocardiography (Mindray DC-N3 ultrasound scanner, Shenzhen Mindray Bio-Medical Electronics Co., Ltd., Shenzhen, China); pulse oximetry (MD300 I device, Beijing Choice Electronic Technology Co., Ltd., Bejing, China); and cytological examination of sputum.

The study on patients was conducted in compliance with the ethical principles of the current version of the Declaration of Helsinki [22] and was approved by the Ethics Committee of the Novosibirsk State Medical University (protocol No. 121, dated 21 November 2019).

### 2.2. Synthesis of Silica Nanoparticles (SiNPs)

SiNPs were synthesized according to a previously described method [23]. The reaction mixture was prepared by sequentially mixing 1 mL 98% ethanol, 30 μL 32% ammonia, and 4.2 μL tetraethoxysilane (TEOS). The final concentrations in the reaction mixture were 0.019 M TEOS and 0.44 M ammonia. The synthesis was carried out at 25 °C for 17 h under constant stirring at 600 rpm. The resulting NPs were collected by centrifugation at 14,000× *g* for 7 min and subjected to three washing steps with 1 mL of ethanol followed by washing with 1 mL of deionized water, using centrifugation under the same conditions for each washing step.

The characteristics of the nanoparticles used in the study were as follows: size by number, 91 ± 1 nm; size by volume, 108 ± 2 nm; size by intensity using dynamic light scattering, 119 ± 2 nm; PDI, 0.04 ± 0.01; Z-Average, 113 ± 1 nm; Zeta potential, −28.6 ± 0.2 mV (Figure S1).

### 2.3. Synthesis of Magnetic Nanoparticles (MNPs)

The synthesis of MNPs was adapted from [24,25]. Briefly, 0.918 g of FeCl_3_·6H_2_O (3.4 mmol) and 0.322 g of FeCl_2_·4H_2_O (1.7 mmol) were dissolved in 40 mL of deionized water and heated at 80 °C for 5 min. After incubation, 7.5 mL of NaOH (2 M) was added to reach pH 11.0 under stirring at 750 rpm and heating at 80 °C for 15 min. The mixture was cooled to room temperature. The obtained MNPs were collected using a magnet and washed three times with 40 mL of deionized water. Next, 2.3 mg of MNPs was dispersed in 3 mL of 98% ethanol. Then, 75 μL of 32% ammonia and 10.5 μL of tetraethoxysilane (TEOS) were added to the suspension. The final concentrations in the reaction mixture were 0.75 mg/mL MNP, 0.015 M TEOS, and 0.35 M ammonia. The synthesis was carried out at 25 °C for 17 h under constant stirring at 600 rpm. The resulting Fe_3_O_4_@SiO_2_ NPs were collected by centrifugation at 14,000× *g* for 7 min and subjected to three washing cycles: first with 1 mL of ethanol, then with 1 mL of deionized water.

The characteristics of the obtained nanoparticles used were as follows: size by number, 246 ± 3 nm; size by volume, 291 ± 11 nm; size by intensity using dynamic light scattering, 284 ± 9 nm; PDI, 0.07 ± 0.02; Z-Average, 268 ± 6 nm; Zeta potential, −20.1 ± 0.1 mV (Figure S2).

### 2.4. Mice

Ten–twelve-week-old C57Bl6 female mice were obtained from the vivarium of Knorre Institute of chemical biology and fundamental medicine SB RAS (Novosibirsk, Russia). Mice were housed in plastic cages under standard daylight conditions (12/12 h light/dark cycle). Water and food were supplied ad libitum. All animal procedures were conducted in strict compliance with the guidelines for the proper use and care of laboratory animals (ECC Directive 2010/63/EU) and ARRIVE guidelines 2.0 [26,27]. The experimental protocols were approved by the Committee on the Ethics of Animal Experiments of the Institute of Cytology and Genetics SB RAS (protocol No. 131, dated 19 October 2022). All intranasal administrations were performed under isoflurane anesthesia using a gas mixture containing 3% isoflurane and  97% air with a flow rate of 2 L/min. Terminal sacrifice and sacrifice of mice withdrawn from the experiment was also performed under isoflurane anesthesia.

### 2.5. SiNP Administration Protocols

Two experimental protocols of SiNP administration were applied. In the first protocol, SiNPs were administered intranasally to mice at a dose of 1 mg per mouse in 50 µL of saline buffer twice a week. Mice were sacrificed after 4 and 6 weeks of consecutive instillations, resulting in a total of 8 and 12 administrations, respectively. In addition, five mice subjected to the 6-week administration protocol were allowed to survive for a further 6 weeks without intervention (follow-up). Thus, in the first protocol mice were divided into four groups: healthy (untreated) (*n* = 5), 4 weeks administration (*n* = 5), 6 weeks administration (*n* = 5), follow-up (*n* = 5).

In the second protocol, SiNPs were administered intranasally to mice at doses of 0.5 and 0.2 mg per mouse in 50 µL of saline buffer twice a week. The control groups were untreated and mice administered saline buffer alone. Mice were sacrificed after 2 and 4 weeks of consecutive instillations, resulting in a total of 4 and 8 administrations, respectively. Five mice subjected to the 4-week administration protocol were allowed to survive for a further 6 weeks without intervention (follow-up). Thus, in the second protocol, mice were divided into ten groups: healthy (untreated) (*n* = 5); saline—2 weeks administration (*n* = 5), 4 weeks administration (*n* = 5), follow-up (*n* = 5); 0.2 mg—2 weeks administration (*n* = 5), 4 weeks administration (*n* = 5), follow-up (*n* = 5); 0.5 mg—2 weeks administration (*n* = 5), 4 weeks administration (*n* = 5), follow-up (*n* = 5).

At all indicated time points, lung samples were collected for histological analysis and Si quantification. Additionally, in the second series of experiments lymph nodes for histology and Si quantification were also collected. Restricted mean survival time (RMST) was calculated using the «survival» package in the RStudio (v. 2025.09.01 build 401) software.

### 2.6. MNP Administration Protocol

MNPs were administered intranasally to mice at a dose of 1 mg per mouse in 50 µL of saline buffer twice a week. Mice were sacrificed after 4 and 6 weeks of consecutive instillations, resulting in a total of 8 and 12 administrations, respectively. Thus, mice were divided into three groups: healthy (untreated) (*n* = 5), 4 weeks administration (*n* = 5), and 6 weeks administration (*n* = 5).

Lung samples were collected for histological analysis as well as Fe and Si quantification.

### 2.7. Histology and Morphometry

For the histological study, the specimens of the lungs and lymph nodes were fixed in 10% neutral-buffered formalin (BioVitrum, Moscow, Russia), dehydrated in ascending ethanols and xylols and embedded in HISTOMIX paraffin (BioVitrum, Russia). The paraffin sections (up to 5 μm) were sliced on a Microm HM 355S microtome (Thermo Fisher Scientific, Waltham, MA, USA) and stained with hematoxylin and eosin. The extracellular matrix (ECM) deposition was determined using Van Gieson’s staining.

The intensity of inflammatory changes, including acute granulocytic inflammation, lymphocytic infiltration, and silicotic nodule formation, as well as ECM content were assessed semi-quantitatively using the following scale: 0—none, 1—mild, 2—moderate, 3—severe, and 4—total. Morphometric assessment of airway remodeling was performed by the estimation of bronchial wall thickness-to-diameter ratio (T/D ratio). In this case, the thickness (T) was measured from the mucosa to the adventitia of the bronchus, and the diameter (D) was calculated using the circumference formula: D = P/π, where P is the perimeter of the bronchus measured along the basement membrane. Approximate mean volumes of lymph nodes were calculated using scans of stained lymph node sections as follows: V = (D × d^2^)/2, where D is the longest diameter of the node section and d is the shortest diameter of the node section perpendicular to D. All measurements were performed using Adobe Photoshop CS3 software. For each parameter, five to ten random fields were studied in each specimen forming 25–50 random fields for each group of mice.

All the images were examined and scanned using Axiostar Plus microscope equipped with Axiocam MRc5 digital camera (Zeiss, Oberkochen, Germany) at magnifications of ×100 (lymph nodes) and ×200 (lungs and lymph nodes).

### 2.8. Si and Fe Quantification

The element content in the lungs (Si and Fe) and lymph nodes (Si) was determined by inductively coupled plasma atomic emission spectrometry (ICP-AES). The organs under investigation were collected, then frozen and stored at −20 °C. The samples were proceed according to a previously described protocol [28,29]. Briefly, samples were dissolved in concentrated HNO_3_ solution at 90 °C, and then it was allowed to stand for 30–60 min until a clear solution was obtained. The resulting mixture was diluted by ultra-pure water (with a resistivity of more than 18 MOm/cm). The determination of Si and Fe concentrations was performed using an iCap-6500 Duo high-resolution ICP atomic emission spectrometer (Thermo Fisher Scientific, Waltham, MA, USA) with a SeaSpray pneumatic nebulizer (Glass Expansion, Melbourne, Australia) and cyclone-type Tracey spray chamber (Glass Expansion, Australia). The analytical signal was recorded in a plasma axial survey for two analytical lines, B 249.678 nm and B 249.773 nm. Signals were detected at parameters recommended by the manufacturer: rf generator power, 1150 W; Ar nebulizier flow rate, 0.7 L/min; auxiliary Ar flow rate, 0.5 L/min; and cooling argon flow rate, 12 L/min. The result was obtained by averaging the data from the two analytical lines. The measurement error was ≤10%.

### 2.9. Analysis of Immune Cell Populations in the Peripheral Blood

Peripheral blood was collected from the retro-orbital sinus of the mice using EDTA-coated tubes to prevent blood coagulation. Red blood cells were lysed with lysis buffer (0.15 M NH_4_Cl, 10 mM NaHCO_3_, and 0.1 mM EDTA) for 5 min at room temperature followed by washing with PBS buffer. Blood cells were resuspended in staining buffer (PBS supplemented with 2% FBS) for subsequent analysis.

The phenotype of immune cells was assessed by flow cytometry. 1 × 10^6^ peripheral blood cells in 100 µL of staining buffer were used in every test. First, cells were Fc-blocked with anti-mouse CD16/CD32 IgG antibodies (#553142, clone 2.4G2, BD Biosciences, San Diego, CA, USA) according to the manufacturer’s recommendations. To characterize immune cells, the following anti-mouse monoclonal antibodies were used: anti-CD45-FITC (#553080, clone 30-F11, BD Biosciences, USA), CD11b-PerCP (#E-AB-F1081UF, clone M1/70, Elabscience, Houston, TX, USA), Ly6C-PE (#ab25572, clone HK1.4, Abcam, Cambridge, UK), Ly6G-violetFluor 450 (#ab253070, RB6-8C5, Abcam, UK), CD3-PE-Cy7 (#E-AB-F1013H, clone 17A2, Elabscience, USA), CD4-PE (#E-AB-F1097D, clone GK1.5, Elabscience, USA), CD8- Brilliant Violet 785 (#284571, clone 53-6.7, Sony, San Jose, CA, USA), and CD25-APC (#E-AB-F1102E, clone PC-61.5.3, Elabscience, USA). A detailed description of extracellular and intracellular staining is given in [30]. The cells were stained with antibodies (1:100) for 30 min at room temperature, followed by staining with secondary polyclonal anti-mouse IgG-AF488 antibodies (1:1000, #ab150077, Abcam, UK) for 30 min at room temperature in the dark, washed twice with staining buffer, and then fixed in 2% formaldehyde in PBS.

Flow cytometry measurements were performed using a NovoCyte 3000 flow cytometer (ACEA Biosciences, San Diego, CA, USA), at least 10,000 events were acquired for each sample. The data were processed using NovoExpress software v. 1.1.0 (ACEA Biosciences, USA). Gating strategies for cell population are shown in (Figure S3).

### 2.10. Statistics

For animal studies, statistical analyses were performed using GraphPad Prism version 10.4.0 using the Mann–Whitney U test or the Kruskal–Wallis H test. Differences were considered statistically significant at *p* ≤ 0.05. For patient cohorts, qualitative indicators were compared using the χ^2^ test. Quantitative indicators were compared using the Mann–Whitney U test for comparisons between two groups and the Kruskal–Wallis test for comparisons between more than two groups. Differences were considered statistically significant at *p* ≤ 0.01.

Data are presented as the mean ± standard error of the mean (SEM).

## 3. Results

### 3.1. Comparative Clinical Characterization of the O-COPD Patient Cohort

To elucidate the clinical and functional trajectory of occupational COPD (O-COPD), we performed a comprehensive characterization of a prospectively enrolled cohort. This investigation was designed to compare patients with O-COPD against distinct groups: workers exposed to similar occupational hazards but remaining apparently healthy, patients with conventional tobacco-smoking COPD, and unexposed healthy volunteers. By integrating demographic profiling, pulmonary function tests, imaging data, and cytological analysis, we aimed to delineate the unique pathophysiological signature of O-COPD and to assess how the specific composition of occupational nanoparticles shapes disease phenotype.

#### 3.1.1. Demographic and Baseline Clinical Characteristics of the Study Cohort

The prospective cohort clinical study included 50 patients with O-COPD exposed to multicomponent industrial aerosols in line of their job, 50 apparently healthy individuals working under similar conditions, and 50 patients with tobacco-smoking COPD without occupational health risks. The main clinical characteristics of the subjects at the time of enrollment are presented in Table S2. The median age of patients in the O-COPD group was 58 (54; 63) years, in the healthy workers group 57 (54; 61.5) years, and in the tobacco-smoking COPD group 60 (55; 62) years, *p* = 0.31. The number of men and women in the O-COPD group was 47 (94%) and 3 (6%), 46 (92%) and 4 (8%) in the healthy workers group, and COPD group 46 (92%) and 4 (8%) in the tobacco-smoking COPD group, *p* = 0.25. The study also included 50 conditionally healthy volunteers without exposure to industrial aerosols, who constituted the control group. The median age in this group was 57 (54; 59) years, with 46 (92%) men and 4 (8%) women. The participants in the control group had no detectable diseases or pathological conditions identified by standard methods and had no occupational health risks.

Smoking history was similar for individuals working in hazardous industrial conditions, tobacco smokers and the conditionally healthy volunteers: pack-years index and duration of smoking were approximately 16 (*p* = 0.15) and 25 years (*p* = 0.45) for all studied groups, respectively (Table S2). Workers exposed to aerosols, both those with O-COPD and the conditionally healthy workers, were comparable in terms of length of occupation under hazardous industrial conditions: 23 (19; 26) and 21 (18; 26) years, respectively (*p* = 0.25) (Table S2). Patients with O-COPD were comparable to patients with tobacco-smoking COPD in terms of COPD duration: 12 (8; 15) and 14 (10; 16) years, respectively (*p* = 0.52). Thus, all groups were matched for demographic indicators, smoking history, and COPD duration.

#### 3.1.2. Distinctive Clinical and Functional Features of O-COPD Compared to Tobacco-Smoking COPD

**Figure 1:**
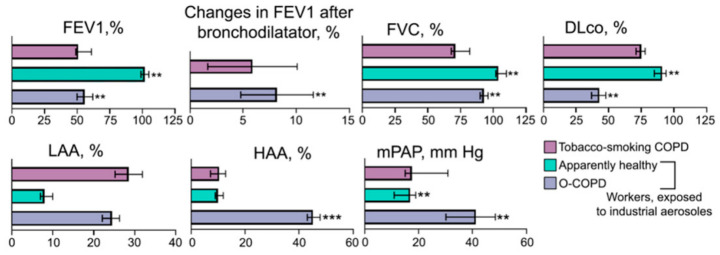
Phenotypic characteristics of occupational COPD (O-COPD) patients included in the study. FEV1—forced expiratory volume in 1 s, %. FVC—forced vital capacity, %. DLco—diffusion capacity for carbon monoxide, %. LAA—low attenuation area, %. HAA—high attenuation area, %. mPAP—mean pulmonary artery pressure, mm Hg. Tobacco-smoking COPD, *n* = 50; apparently healthy, *n* = 50; O-COPD, *n* = 50. Error bars denote upper and lower limit of parameters, except for «Changes in FEV1 after bronchodilatator», where error bars denote standard deviation. ** *p* ≤ 0.01, *** *p* ≤ 0.001.

Occupational COPD significantly differed from tobacco-smoking COPD by more pronounced restrictive impairments (forced vital capacity (FVC) was 75.3 ± 4.1% and 93.8 ± 3.1%, respectively, *p* = 0.01) with less severe bronchial obstruction (forced expiratory volume in 1 s (FEV1) was 56.5 ± 5.4% and 51 ± 6.1%, respectively, *p* = 0.01), as well as by more significant reduction in lung diffusion capacity (DLco values were 42.8 ± 5.2% and 75.2 ± 3.5%, respectively, *p* = 0.01) (Figure 1, Table S2). Chest computed tomography (CT) identified the “emphysema-pulmonary fibrosis” phenotype in 90% (*n* = 45) of O-COPD patients and 48% (*n* = 24) of tobacco-smoking COPD patients, *p* = 0.02. It should be noted that the emphysema index (low attenuation area during CT, LAA, %) was similar for these groups, being 24.5 ± 5.3% for O-COPD patients and 28.5 ± 4.4% for tobacco-smoking COPD patients, whereas the pulmonary fibrosis index (high attenuation area during CT, HAA, %) was significantly higher in the O-COPD group (45.2 ± 7.1%) compared to the tobacco-smoking COPD group (10.5 ± 3.2%), *p* = 0.001 (Figure 1, Table S2). Signs of pulmonary hypertension were also more pronounced in patients with O-COPD compared to patients with tobacco-smoking COPD: the mean pulmonary artery pressure (mPAP) for these groups was 41.3 ± 6.1 and 17.6 ± 5.3 mm Hg, respectively, *p* = 0.01 (Figure 1, Table S2). Cytological examination of sputum in O-COPD patients revealed a predominantly paucigranulocytic airway inflammation phenotype. In contrast, tobacco-smoking COPD patients were characterized mostly by neutrophilic inflammation (Table S2). Exercise tolerance, assessed by the six-minute walking test, and symptom severity according to the COPD Assessment Test (CAT) score did not differ between the O-COPD and tobacco-smoking COPD groups (Table S2).

Thus, O-COPD differed from tobacco-smoking COPD by higher severity of restrictive impairments, reduced lung diffusion capacity, more pronounced pulmonary hypertension, presence of pulmonary fibrosis, and paucigranulocytic airway inflammation phenotype.

#### 3.1.3. Influence of Occupational Nanoparticle Composition on O-COPD Phenotype

The examined O-COPD patients were employed at a machine-building enterprise (occupations: batchman, molder, chipper, grinder, foundry worker, welder). Assessment of nanoparticles in industrial aerosols with their morphology and size estimation was carried out previously [31]. The matrix elemental composition of individual particles was determined by energy-dispersive spectrometry, revealing the presence of Al, Cr, Cu, Fe, S, Sn, W, Zn, and Zr. The total elemental composition of the solid aerosol particles determined by inductively coupled plasma atomic emission spectrometry (ICP-AES) was represented by Al, B, Ba, Ca, Cu, Fe, K, Mg, Mn, Na, Ni, P, S, Si, Sr, and Zn. Particle concentrations in patients’ workplaces ranged from 5 to 635 µg/m^3^ [32,33]. Technological operations performed by batchmen, molders, chippers, and grinders are associated with dust emissions containing from 10% to 70% silicon dioxide (highly fibrogenic dust), with mass concentration of silica nanoparticles being the highest (0.035 µg/mL), and metal nanoparticles being minimal. As for the adverse occupational factors for foundry workers and welders, these are metal fumes (compounds of aluminum, iron, lead, chromium) and welding fumes (compounds of iron, chromium, carbon monoxide), respectively, with higher concentration of metal nanoparticles (Al—0.0031 µg/mL, Fe—0.0042 µg/mL, Cr—0.00021 µg/mL) and minimal concentration of silica nanoparticles.

The apparently healthy workers exposed to occupational hazards were matched to the patient group by job titles. The baseline data of O-COPD patients after stratification by the predominant chemical composition of incidental nanoparticles in the industrial aerosol are presented in Table S3. The groups were comparable in terms of sex, age, COPD duration, and did not differ in occupational exposure duration or smoking history (Table S3).

**Figure 2:**
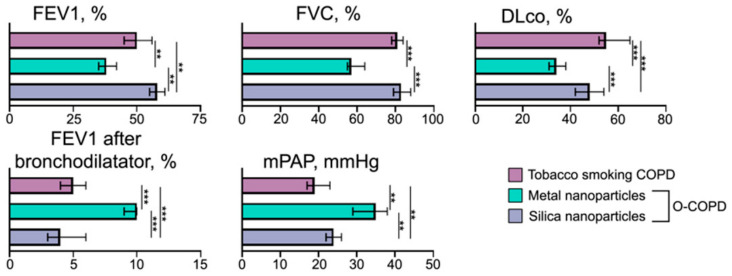
Phenotypic characteristics of occupational COPD (O-COPD) patients depending on chemical composition of industrial aerosols. FEV1—forced expiratory volume in 1 s, %. FVC—forced vital capacity, %. DLco—diffusion capacity for carbon monoxide, %. mPAP—mean pulmonary artery pressure, mm Hg. Tobacco-smoking COPD, *n* = 50; O-COPD, exposure to metal nanoparticles, *n* = 26; O-COPD, exposure to silica nanoparticles, *n* = 24. Error bars denote upper and lower limits of parameters. ** *p* ≤ 0.01, *** *p* ≤ 0.001.

During development of O-COPD under exposure to aerosols containing metal nanoparticles, lung ventilation function was characterized by greater severity of bronchial obstruction, higher annual rate of FEV1 decline, and a reduction in lung diffusion capacity (Figure 2, Table S3). This group also exhibited the largest increase in FEV1 after bronchodilator inhalation, indicating heightened bronchial reactivity. In contrast, in patients with O-COPD developed due to exposure to aerosols containing silica nanoparticles, reduction in lung diffusion capacity was observed alongside less severe bronchial obstruction. The rate of FEV1 decline was the lowest in this group (Figure 2, Table S3). Mean pulmonary artery pressure (mPAP) was highest in patients exposed to metal nanoparticles, whereas patients exposed to silica nanoparticles exhibited a better clinical profile (Figure 2, Table S3). Sputum analysis revealed predominantly eosinophilic inflammation in O-COPD patients exposed to metal nanoparticles, and paucigranulocytic inflammation in those exposed to silica nanoparticles (Table S3).

A univariate regression analysis revealed associations between the concentration of nanoparticles in the workplace air and the phenotypic characteristics of O-COPD (Table 1). The concentration of both metal and silica nanoparticles assessed previously [32,33] was associated with FEV1 and DLco (Table 1). At the same time, the concentration of metal nanoparticles was significantly associated with the emphysema index (LAA, %) and eosinophilic inflammation phenotype, while the mass concentration of silica nanoparticles was associated with the extent of pulmonary fibrosis (HAA, %) and the paucigranulocytic inflammation phenotype (Table 1). Additionally, the multivariate analysis demonstrated the influence of the mass concentration of metal nanoparticles on the LAA, % (B = 1.3, *p* = 0.001), the eosinophilic inflammation phenotype (B = 2.3, *p* = 0.001), and FEV1 (B = −1.4, *p* = 0.002). The concentration of silica nanoparticles was associated with DLco (B = −1.7, *p* = 0.001) and the paucigranulocytic airway inflammation phenotype (B = 1.9, *p* = 0.001).

Finally, the clinical phenotyping of O-COPD patients revealed two different disease endotypes that correlate with the type of inhaled nanoparticles: individuals exposed primarily to metal nanoparticles were characterized by severe airflow limitation, pronounced bronchodilator reversibility, and eosinophilic inflammation. In contrast, silica nanoparticle-exposed patients exhibited better preserved spirometry, minimal bronchodilator response, and a predominance of paucigranulocytic inflammation, accompanied by a progressive fibrotic phenotype (higher HAA, % in lung CT). Regression analyses (Table 1) substantiated these clinical patterns. Thus, associations between the chemical composition of nanoparticles in industrial aerosols and O-COPD phenotype confirm the formation of specific inflammation patterns apparently mediated by diverse mechanisms with predominant obstructive changes and increased bronchial reactivity upon exposure to metal nanoparticles and fibrotic changes in the lungs upon exposure to silica nanoparticles. The formation of irreversible fibrotic changes in the lungs after exposure to silica nanoparticles indicates the need for further study of their properties, including development of relevant mouse models.

### 3.2. Establishment of a Nanoparticle-Induced Murine Model of O-COPD

To accurately follow the main pathogenetic and morphological signs of O-COPD, characterized by the steady progression of airway and lung fibrosis resulting in irreversible diminishment of lung function, translational in vivo models are urgently needed. For this purpose, prolonged and repeated exposure to nanometer-sized particles entering the respiratory system via the nasopharyngeal route, similar to human O-COPD cohorts in industrial settings, is necessary. However, the question regarding the chemical composition and dosage of consumed particles as well as detectable time points remains open. Because specific clinical patterns and outcomes of the exposure to industrial aerosols containing silica and metal nanoparticles were identified in the patient cohort, the murine model of O-COPD nanoparticles of these particular compositions was used.

#### 3.2.1. Protocol 1: 4- and 6-Week Administration of Silica and Magnetic Nanoparticles

**Figure 3:**
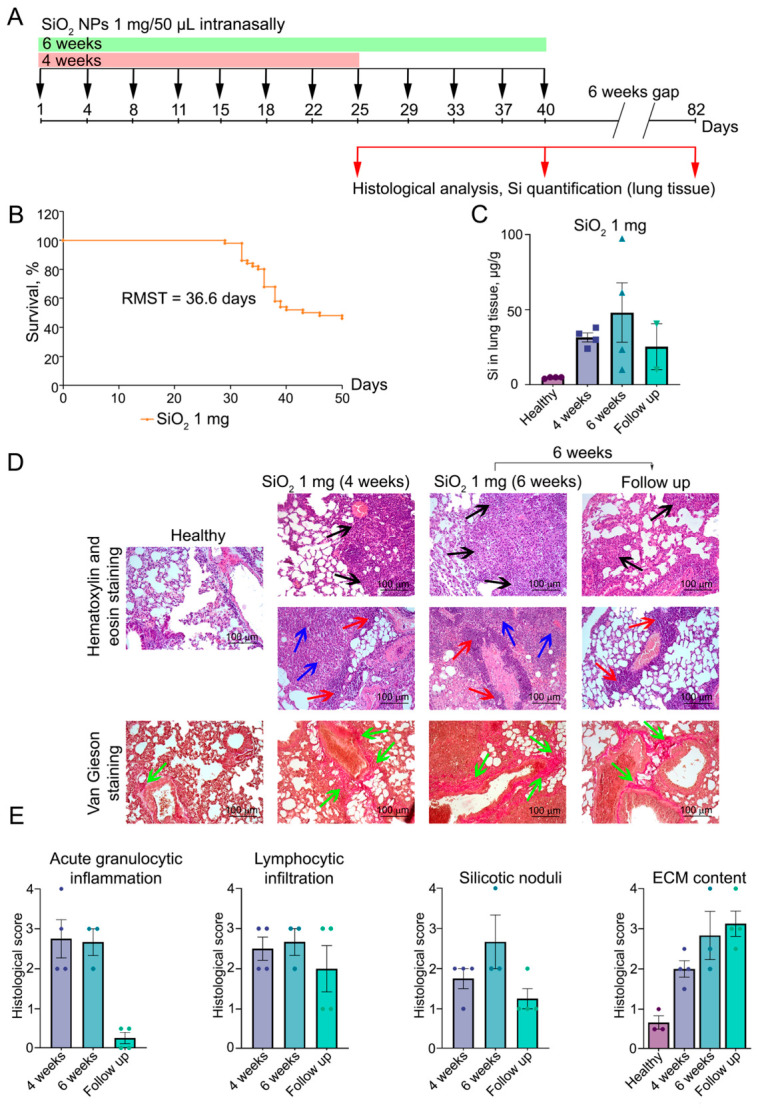
Characterization of the in vivo model of silica nanoparticle (SiNP)-induced pulmonary inflammation and fibrosis. (**A**) Experimental setup. SiNPs were administered intranasally at a dose of 1 mg per mouse twice a week. Mice were sacrificed after 4 and 6 weeks of consecutive instillations. Five mice subjected to the 6-week experiment were allowed to survive for an additional 6 weeks without intervention (follow-up). At all indicated time points, lung samples were collected for histological analysis and Si quantification; *n* = 5 for each experimental group. (**B**) Restricted mean survival time (RMST) of mice after SiNP administration. (**C**) Si content in the lungs of healthy and SiNP-administered mice. (**D**) Representative histological images of the lung sections of healthy and SiNP-administered mice. Hematoxylin and eosin staining (**upper** panels), Van Gieson staining (**bottom** panel). Original magnification ×200. Black arrows indicate silicotic nodules. Red arrows indicate lymphocytic infiltration. Blue arrows indicate acute granulocytic inflammation. Green arrows indicate the deposition of extracellular matrix (ECM) components. (**E**) Histological scoring of morphological changes in the lung tissue. The intensity of acute granulocytic inflammation, lymphocytic infiltration, silicotic nodule formation, and ECM content were assessed semi-quantitatively using the following scale: 0—none, 1—mild, 2—moderate, 3—severe, and 4—total.

**Figure 4:**
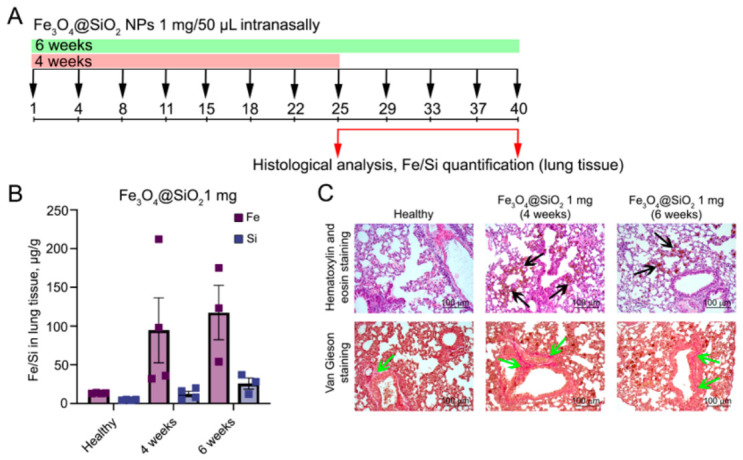
Effects of repeated exposure to magnetic nanoparticles (MNPs). (**A**) Experimental setup. MNPs were administered intranasally at a dose of 1 mg per mouse twice a week. Mice were sacrificed after 4 and 6 weeks of consecutive instillations. Lung samples were collected for histological analysis and Fe/Si quantification; *n* = 5 for each experimental group. (**B**) Fe and Si contents in the lungs. (**C**) Representative histological images of the lung sections of mice after MNP administration. Hematoxylin and eosin staining (**upper** panel), Van Gieson staining (**bottom** panel). Original magnification ×200. Black arrows indicate macrophages with engulfed MNPs, as well as free-lying MNPs. Green arrows indicate the extracellular matrix (ECM) in the lung tissue.

To investigate the primary effects of repeated nanoparticles exposure, silica nanoparticles (SiNPs) represented by silicon dioxide (SiO_2_) or magnetic nanoparticles (MNPs) composed of Fe_3_O_4_ coated with SiO_2_ (Fe_3_O_4_@SiO_2_) were utilized. In protocol 1, mice were subjected to regular instillations of SiNPs or MNPs at a dose of 1 mg per mouse in 50 µL saline buffer, administered twice a week for a total duration of four and six weeks (Figure 3A and Figure 4A). Morphological changes and Si or Fe accumulation in the lungs were assessed at four and six weeks for SiNPs and MNPs, and at six weeks after the final instillation (the follow up endpoint) for SiNPs (Figure 3A and Figure 4A).

The instillation of SiNPs at a dose of 1 mg/mouse twice a week induced severe morbidity, with mouse mortality observed around week 4. By the experimental endpoint, six weeks after the final instillation, only 46% of the mice survived, with a restricted mean survival time (RMST) of 36.6 days (Figure 3B). Administration of MNPs did not cause mortality in experimental animals.

Quantification of element content in the lung tissue shows a progressive accumulation of Si during SiNP administration, with concentration reaching maximal values at the 6-week time point (~50 µg/g of lung tissue). Notably, a certain amount of Si (~25 µg/g) persisted within the lungs even after the 6-week follow-up, indicating the deferred rate of SiNPs clearing from the lung tissue (Figure 3C). Intranasal instillations of MNPs, composing of Fe_3_O_4_@SiO_2_, caused a significant accumulation of Fe in the lung tissue throughout 4- and 6-week administration periods (~200 µg/g), while Si content remained low, reaching ~30 µg/g after 6 weeks of continuous instillations (Figure 4B).

Histological analysis revealed severe inflammatory changes in the lungs of mice exposed to SiNPs. These changes included massive granulocytic infiltration with edema and focal hemorrhage, expressed equally both at four and six weeks of SiNP administration and completely resolved after a 6-week withdrawal period (Figure 3D,E). Furthermore, lymphocyte infiltration including NK cells, CD4^+^ and CD8^+^ T lymphocytes, as well as B lymphocytes were detected primarily around the bronchi and blood vessels, representing a T-cell immune response to SiNPs. The intensity of this process remained at approximately the same level throughout the entire observation period (Figure 3D,E).

An important sign of lung damage from SiNPs is the formation of silicotic nodules, representing clusters of alveolar macrophages (AMs) containing engulfed SiNPs, with proliferating alveolar epithelial type II cells (AECs II) and nascent collagen fibers. However, the presence of massive granulocytic infiltration complicated their assessment and made the prevalence of silicotic nodules quite moderate after 4 and 6 weeks of SiNP administration as well as by the end of the 6-week follow-up period (Figure 3D,E). Nevertheless, the most significant morphological sign of lung damage due to the presence of SiNPs was the development of pulmonary fibrosis with connective tissue proliferation around the bronchi, blood vessels, and within the pulmonary parenchyma. As can be seen from Figure 3D,E, ECM content in the lungs of SiNP-administered mice progressively increased by ~3 to 4-fold compared to healthy animals during the administration period and persisted even after cessation of administration.

MNPs administered at the same concentration and by the same route as SiNPs did not cause inflammatory and fibrotic changes in lung tissue, despite their significant accumulation in lung structures both inside the macrophages engulfing MNPs and those freely lying around the bronchi (Figure 4C).

Thus, repeated (4 and 6 weeks) administration of SiNPs at a dose of 1 mg per mouse resulted in irreversible lung tissue remodeling, primarily manifested by the deposition of ECM components, as well as a persistent lymphocytic response, which remained at a constant level even after a 6-week period without SiNP administration. In addition, the administration of SiNPs according to the indicated scheme caused a severe acute inflammatory response, making it difficult to assess the formation of silicotic nodules, which are a pathognomonic sign of silica-induced lung damage. In contrast, MNPs did not induce such fibrotic and inflammatory changes. To a certain extent, this corresponded to the clinical picture in the patients’ cohort exposed to industrial aerosols containing silica or metal nanoparticles. The contrasting effects of exposure to SiNPs and MNPs may be associated, on the one hand, with the complex composition of industrial aerosols and, on the other hand, with the chemical properties of the synthetic NPs used in this experiment [32,33,34].

#### 3.2.2. Protocol 2: 2- and 4-Week Administration of Silica Nanoparticles

**Figure 5:**
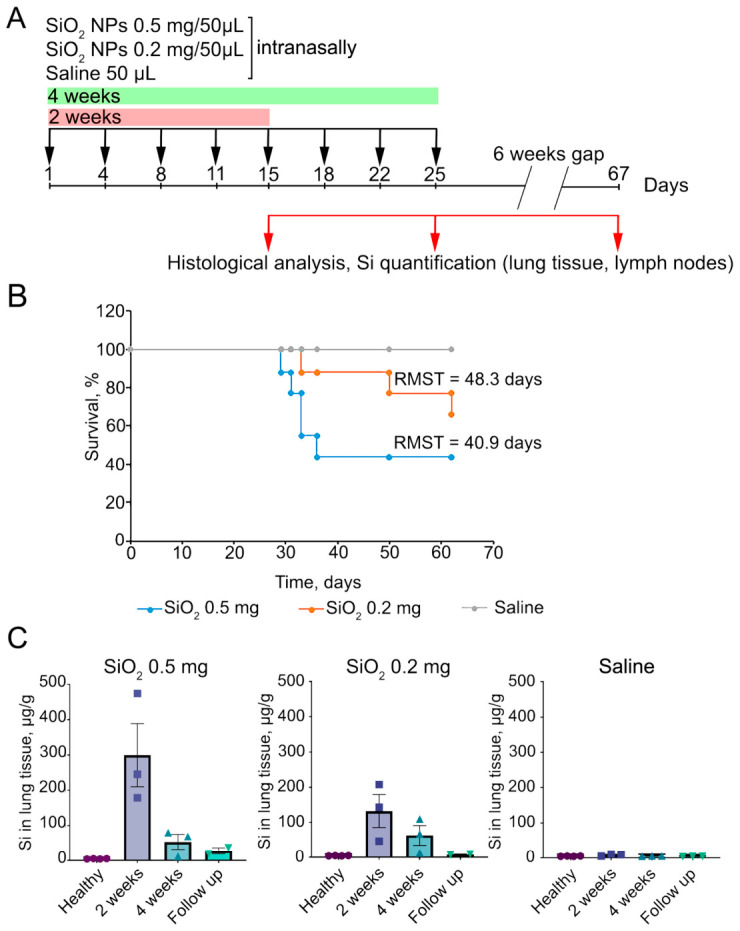
Dose-dependent model of silica nanoparticle (SiNP)-induced pulmonary inflammation and fibrosis. (**A**) Experimental setup. SiNPs were administered intranasally at doses of 0.5 and 0.2 mg per mouse, as well as saline buffer alone as a control, twice a week. Mice were sacrificed after 2 and 4 weeks of consecutive instillations. Five mice administered SiNPs for 4 weeks subjected to the 4-week experiment were allowed to survive for an additional 6 weeks without intervention (follow up). At all indicated time points, lung and lymph node samples were collected for histological analysis and Si quantification; *n* = 5 for each experimental group. (**B**,**C**) Restricted mean survival time (RMST) (**B**) and Si content (**C**) in the lungs of mice after SiNP administration.

Building on the initial model, we established a second protocol to thoroughly characterize the pathological effects of SiNPs. Mice were subjected to intranasal instillations of either 0.5 mg or 0.2 mg of SiNPs in 50 µL saline buffer twice a week for two and four weeks, alongside a saline-only control group (Figure 5A). Lung tissues were collected at the end of the administration periods (at 2 and 4 weeks) and at the follow-up time point (6 weeks after the final instillation) for comprehensive histological analysis and Si quantification (Figure 5A). Additionally, lymph nodes for histological analysis and Si quantification were collected.

Survival analysis revealed a clear dose-dependent effect on mice mortality. The onset of lethality occurred on day 29 for the 0.5 mg group and on day 33 for the 0.2 mg group. By the experimental endpoint, survival rates were 44% for the 0.5 mg dose and 66% for the 0.2 mg dose, with corresponding RMSTs of 40.9 and 48.3 days, respectively (Figure 5B). Quantification of lung Si content demonstrated similar accumulation dynamics for both doses, peaking at two weeks after administration, even in the 4 week administration groups, and declining significantly by the follow-up endpoint (Figure 5C). This analysis confirmed a pronounced dose-dependent effect with the 0.5 mg dose group reaching a significantly higher maximum concentration (~300 µg/g) compared to the 0.2 mg group (~130 µg/g) after 2 weeks of SiNP administrations (Figure 5C). Furthermore, the silica burden in the 0.5 mg group decreased to ~25 µg/g by the follow-up endpoint, and the concentration in the 0.2 mg group returned to the baseline levels observed in healthy controls (<5 µg/g) (Figure 5C).

**Figure 6:**
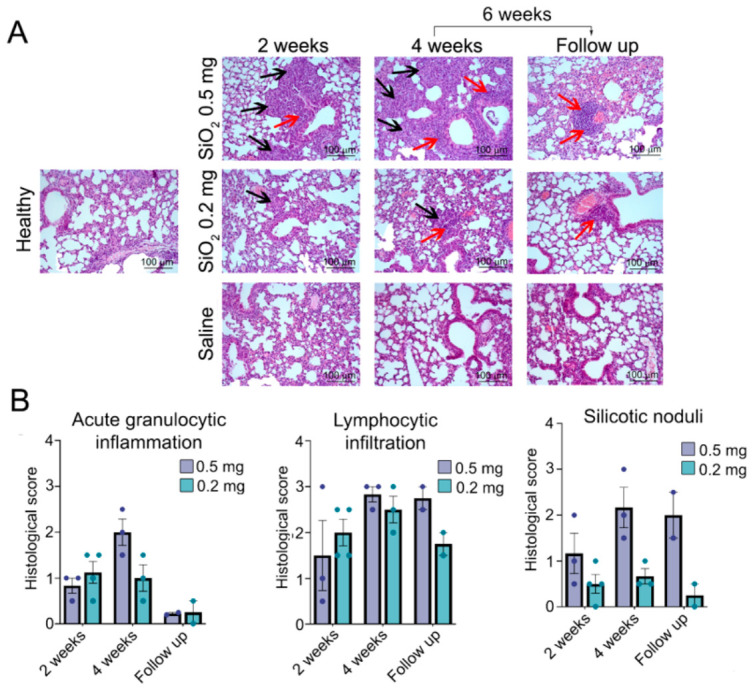
Dose-dependent inflammatory changes in the lung tissue after repeated SiNP administrations. (**A**) Representative histological images of the lung sections of mice with silica-induced inflammation and fibrosis. Hematoxylin and eosin staining. Original magnification ×200. Black arrows indicate silicotic nodules. Red arrows indicate lymphocytic infiltration. (**B**) Histological scoring of morphological changes in the lung tissue of mice was performed according to the scale: 0—none, 1—mild, 2—moderate, 3—severe, and 4—total.

Histological examination of the lungs revealed that administration of SiNPs at lower doses than in the previous experiment (0.5 or 0.2 mg versus 1 mg) did not induce the same severity of acute inflammatory response but stimulated the formation of silicotic nodules and the development of a primary lymphocytic response (Figure 6A,B). The dynamics of these morphological changes were similar at both doses of SiNPs, but their intensity was dose-dependent. Acute granulocytic inflammation and silicotic nodule formation were mild at 2 weeks at both doses, and the difference between these groups at this point was statistically insignificant (Figure 6A,B); however, after 4 weeks of continuous exposure, the intensity of granulocytic infiltration and silicotic nodule formation increased in the 0.5 mg group while remaining unchanged in the 0.2 mg group. Withdrawal of SiNP instillations almost completely halted acute granulocytic inflammatory changes in both groups but did not reduce the formation of silicotic nodules in the 0.5 mg group (Figure 6A,B). The dynamics and intensity of lymphocytic infiltration around the bronchi and vessels were similar in both groups, peaking at 4 weeks after SiNP administration (Figure 6A,B).

**Figure 7:**
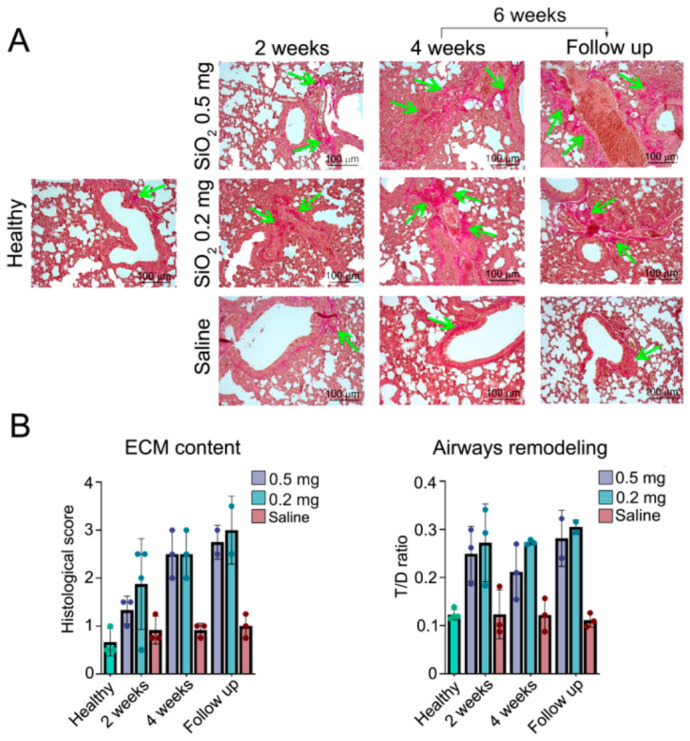
Fibrotic changes in the lung tissue after repeated SiNP administrations. (**A**) Representative histological images of the lung sections of mice with silica-induced inflammation and fibrosis. Van Gieson staining. Original magnification ×200. Green arrows indicate the deposition of extracellular matrix (ECM) components. (**B**) Histological scoring of ECM content (**left** panel) in the lung tissue of mice was performed according to the scale: 0—none, 1—mild, 2—moderate, 3—severe, and 4—total. Morphometric assessment of airway remodeling (**right** panel) was performed by the calculation of bronchial wall thickness-to-diameter ratio (T/D ratio).

However, the most interesting observations concern fibrotic changes in the lungs. Regardless of the SiNP dose administered to the mice, we observed a progressive ~4-fold increase in the ECM content in the lungs at 4 weeks after SiNP administration compared to healthy mice, which remained at a high level even after discontinuation of SiNP instillations (Figure 7A,B).

It should be noted that the intensity of fibrotic changes was approximately the same in experimental protocol 1 and protocol 2, regardless of the dose of administered nanoparticles, and there was an increase in the intensity over the time (Figure 3D,E and Figure 7A,B).

Additionally, due to the low severity of peribronchial acute granulocytic infiltration when using lower doses of SiNPs in protocol 2, histological assessment of airway structure revealed key features of airway remodeling, characterized by cellular and extracellular changes in airways, including goblet cell hyperplasia, smooth muscle cell proliferation, and fibroblast/myofibroblast accumulation with subepithelial fibrosis formation. In our case, these changes were more typical for small airways than for large ones. Morphometric assessment of airway remodeling by calculating the bronchial wall thickness-to-diameter ratio (T/D ratio) revealed that administration of SiNPs at both 0.5 and 0.2 mg per mouse resulted in a 2–2.8-fold increase in this parameter compared to mice treated with saline at the corresponding time points (Figure 7B). Furthermore, a stable sustaining of the T/D ratio over time was noted for both doses, even after cessation of SiNP administration (follow-up).

Repeated intranasal instillations of saline did not have any negative effects on the lungs (Figure 6 and Figure 7).

Thus, the administration of SiNPs in lower doses did not cause severe inflammatory reaction in the lungs, but provoked connective tissue expansion that was no different from those observed when using higher doses. The optimal observation time point should be considered 4 weeks, since the balance in the development of both inflammatory changes and fibrotic changes can be detected.

#### 3.2.3. Lymph Node and Blood Immune Cell Profiles After 2- and 4-Week Administration of Silica Nanoparticles

**Figure 8:**
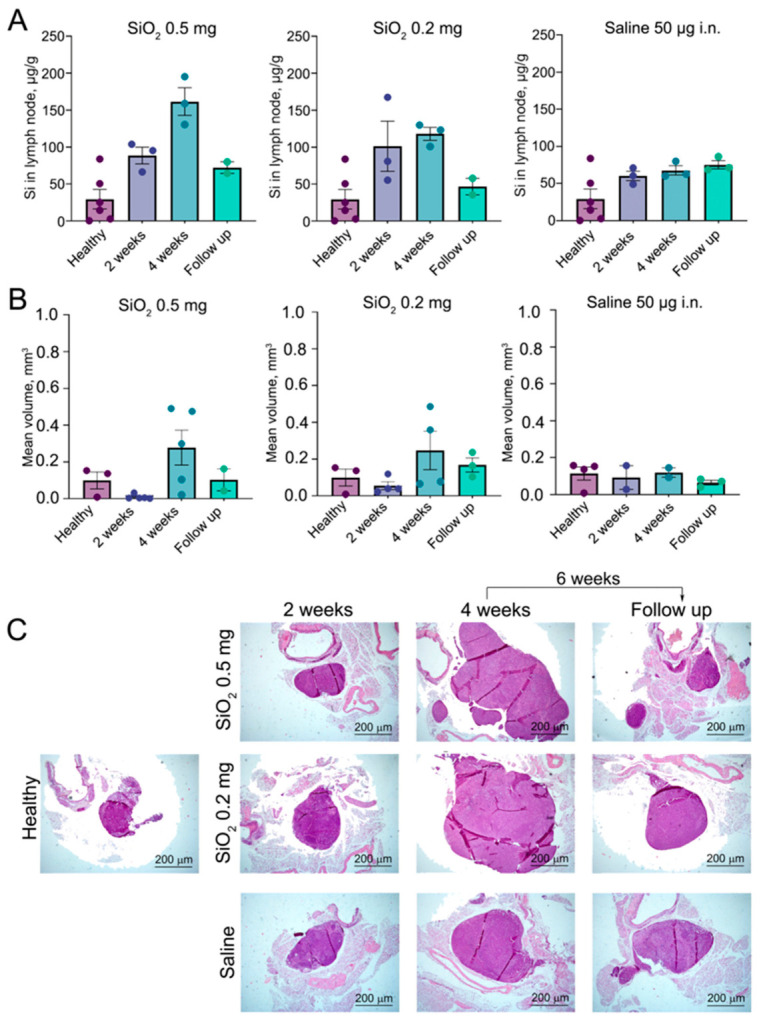
Changes in lymph nodes following silica nanoparticle (SiNP) administration. (**A**–**C**) Si content (**A**), mean volume (**B**) and morphological changes (**C**) of the lymph nodes of mice with SiNP-induced pulmonary inflammation and fibrosis. Hematoxylin and eosin staining. Original magnification ×100.

Due to the fact that 2 weeks of administration of SiNPs caused greater accumulation of Si in the lungs than 4 weeks of administration, we attempted to analyze the clearance of Si from lung tissue by assessing their content as well as the structure of draining lymph nodes. As shown in Figure 8A, Si accumulation in the lymph nodes peaked at 4 weeks of administration, while its levels in the lungs decreased at this time point despite continued administration apparently due to the activation of the lymph node clearance function, which removed SiNPs from the lungs. The observed behavior of lymph node size, which decreased after 2 weeks of SiNP instillations and increased significantly by 2.8 times at the 4-week time point compared to their size in healthy mice (Figure 8B,C), can be associated both with the direct accumulation of SiNPs in the lymph nodes and their reactive hyperplasia in response to interventions, confirming our hypothesis to some extent. Moreover, the changes observed in the lymph nodes were almost independent of the dose of the administered NPs.

Histologically, the lymph nodes of healthy mice and mice treated with saline had a typical structure and consisted of a cortex composed of lymphoid follicles without germinal centers, paracortex, and medulla with medullary cords and sinuses (Figure S4). Administration of SiNPs at both 0.5 and 0.2 mg per mouse induced the development of reactive hyperplasia, which became most pronounced by week 4 of sequential instillations and was manifested by follicular hyperplasia with the formation of germinal centers, as well as the appearance of vacuolated histiocytes that phagocytized the nanoparticles and, in some cases, formed granulomas (Figure S4). Discontinuation of SiNP administration caused a reversal of follicular hyperplasia, but with a decrease in lymph node cellularity and the presence of granuloma-like histiocyte clusters (Figure S4).

Despite acute granulocytic inflammation in the lung tissue throughout the four weeks of nanoparticle administration, along with a lymphoid component contributing to pulmonary inflammatory changes and eventual lymph node involvement in SiNP elimination, we observed no corresponding alterations in systemic levels of myeloid cells or T cells compared to healthy mice (Figure S5). Populations of monocytes, granulocytes, and T lymphocytes in the peripheral blood of SiNP-treated mice remained unchanged (with some tendency of regulatory T-cell expansion) at both doses during the experiment (Figure S5). Together, these findings indicate that the observed immunological effects remain localized without systemic progression, and that the immunological changes induced by SiNPs may be reversible.

Multiple intranasal administrations of saline did not cause any effects on lymph node structure or immune cell profile in the peripheral blood (Figure 8 and Figure S5).

Thus, the draining function state of regional lymph nodes play an important role in clearing the lungs of nanoparticles; however, their accumulation in the lung structures, causing a local inflammatory and remodeling response, does not have a systemic effect on the immune cell profile in the peripheral blood.

## 4. Discussion

Occupational chronic obstructive pulmonary disease (O-COPD) represents a distinct and severe clinical phenotype caused by the chronic inhalation of hazardous materials under industrial conditions [35]. Unlike tobacco-smoking COPD, which is characterized primarily by airflow obstruction and emphysema, O-COPD presents a mixed pathology featuring pronounced restrictive lung function impairment, severe gas exchange deficit, and progressive pulmonary fibrosis [36,37]. The duration of exposure is a critical determinant of disease, with substantial evidence linking longer occupational exposure to both increased risk of developing COPD [38,39] and greater disease severity [40]. In our O-COPD patients’ cohort, a median exposure of 23 years was associated with significant decline in lung function, including reduced forced vital capacity (FVC) and severely impaired lung diffusion capacity (DLco). However, irreversible changes in the lungs, according to our data, were dependent on the composition of the industrial aerosols affecting patients with O-COPD.

Our clinical analysis clearly shows how the composition of occupational aerosols dictates the disease phenotype. Exposure to metal nanoparticles was associated with the obstructive phenotype, characterized by the lowest FEV1 (38% predicted) and the most rapid annual FEV1 decline (71 mL). In contrast, a similar duration of exposure to silica nanoparticles was linked to a distinctly different profile: better-preserved FEV1 (58% predicted) and a slower rate of decline (59 mL), but a significantly more compromised lung structure accompanied by a progressive fibrotic phenotype (higher HAA, % in lung CT). This indicates that long-term silica nanoparticle exposure drives a predominantly restrictive, fibrotic pathophysiology that severely impairs gas exchange, whereas metal fumes accelerate obstructive ventilatory decline. The regression analyses further confirmed these findings, linking metal nanoparticle concentration to the emphysema index (LAA, %) and eosinophilic inflammation, and silica nanoparticle concentration to the fibrosis index (HAA, %) and paucigranulocytic inflammation.

The integrated analysis of clinical and experimental data elucidates the specific pathogenesis of nanoparticle-induced O-COPD. Analysis of the patient cohort confirmed that O-COPD is a distinct disease entity, characterized by a predominant restrictive and fibrotic phenotype, contrary to the pathogenetic mechanisms of tobacco-smoking COPD which leads primarily to obstructive alterations in the lungs [41]. We confirmed that O-COPD-affected patients exhibited significantly lower FVC, severely impaired DLco, and a greater high attenuation area (HAA, %) on CT scans—findings indicative of parenchymal fibrosis. This clinical profile was strongly associated with workplace exposure to aerosols containing silica nanoparticles, confirming their role as a primary etiological agent driving fibrotic lung remodeling in contrast to aerosols containing metal nanoparticles, which cause predominantly increased reactivity changes. These findings in the patient cohort are supported by our experimental animal studies (pronounced fibrotic changes in the lungs upon administration of silica nanoparticles regardless of the concentration used and the absence of similar irreversible changes upon administration of magnetic nanoparticles) and studies by other authors concerning both metal and silica nanoparticles. For example, Kim et al. demonstrated pulmonary clearance and biotransformation of ferric oxide nanoparticles persisting in the lungs without overt toxicity [42], while Moen et al. postulate that nano- and micron-sized iron oxides are often considered to be nontoxic even if very high and prolonged inhalation exposures might result in disease [43]. It has been reliably shown that silica particle exposure causes both inflammatory and fibrotic changes in the lungs of laboratory animals [44,45] and in workers engaged in specific abrasive blasting operations [46,47].

To recapitulate this specific pathology, we designed a murine model of O-COPD based on repeated intranasal SiNP instillations. This approach differs significantly from traditional models that rely on elastase or lipopolysaccharide administrations, which primarily generate emphysema and inflammation but fail to produce persistent airway remodeling and/or pulmonary fibrosis, which is a central hallmark of O-COPD [48]. Moreover, our model of consecutive intranasal low-dose administrations using nano-sized particles diverges from classic single high-dose silicosis models utilizing silica particles with different size ranges that are often administered intratracheally [44,49,50,51], and our model clearly reflects the chronic continuous exposure that O-COPD patients are usually affected by [52]. A significant finding from this model was the dissociation between acute inflammatory responses and fibrotic progression. While mortality and granulocytic infiltration showed clear dose-dependence, the development of extracellular matrix (ECM) deposition and bronchial wall thickening was remarkably dose-independent. Fibrosis progressed and persisted even at lower doses of SiNPs (0.2–0.5 mg per mouse) and continued to evolve after exposure cessation, mirroring the self-sustaining and irreversible nature of the disease observed in patients.

**Figure 9:**
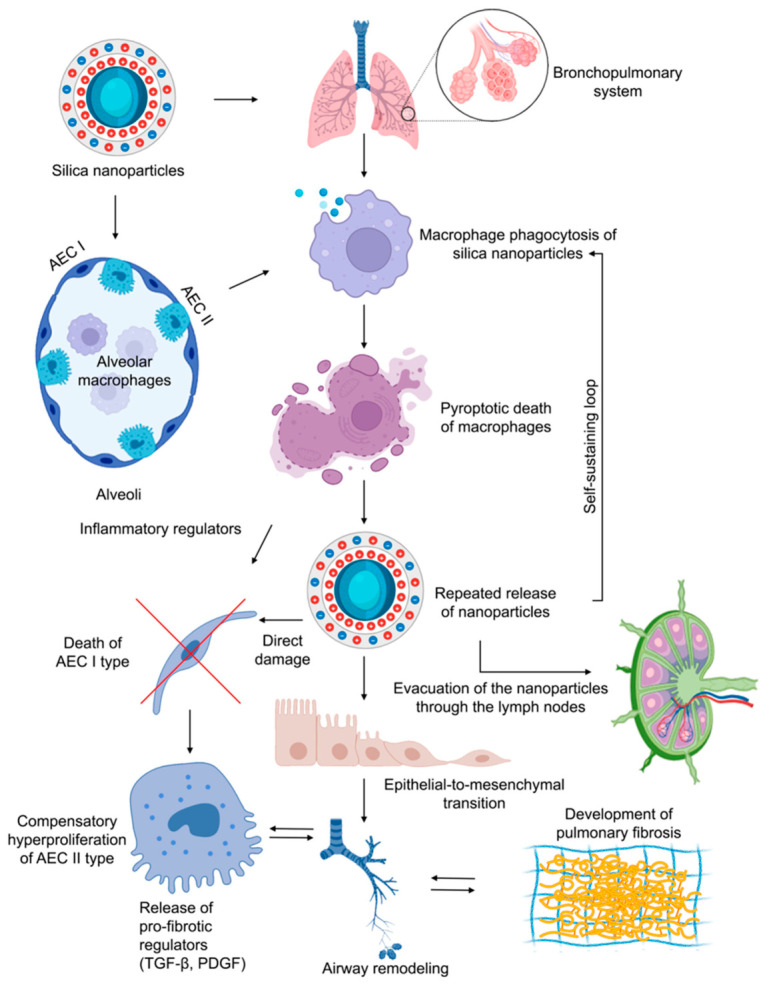
Pathogenetic cascade of silica-induced airway remodeling and pulmonary fibrosis (created in BioRender). Uptake of SiNPs by alveolar macrophages leads to lysosomal damage, inflammasome activation, and pyroptotic cell death. Released nanoparticles are subsequently captured by newly recruited macrophages, perpetuating a self-reinforcing cycle. SiNP-induced injury to alveolar epithelial type I cells (AEC I) triggers hyperproliferation of type II cells (AEC II) and release of pro-fibrotic mediators. This activates fibroblast-to-myofibroblast transition and epithelial–mesenchymal transition, driving airway remodeling and pulmonary fibrosis.

The pathogenesis of this steadily progressing lung fibrosis can be traced to a biochemical cascade initiated by SiNP phagocytosis. Uptake by alveolar macrophages leads to lysosomal damage, inflammasome activation, and pyroptotic cell death (Figure 9). Released nanoparticles are subsequently captured by newly recruited macrophages, perpetuating a self-reinforcing cycle of cell death and inflammation. Concurrently, SiNP-induced injury to alveolar epithelial type I cells (AEC I) triggers hyperproliferation of type II cells (AEC II) and the sustained release of pro-fibrotic mediators such as TGF-β and PDGF [53]. This activates fibroblast-to-myofibroblast transition and epithelial–mesenchymal transition processes, driving aberrant epithelial repair, airway remodeling, and pulmonary fibrosis [54,55].

The model also illuminated a dynamic clearance pathway, with SiNPs accumulating in lung-draining lymph nodes, indicating that after some time the injuring factor is evacuated from the lungs, but the self-sustaining nature of fibrosis means that removal of SiNPs does not lead to reduction in fibrotic tissue that has already formed (Figure 9).

**Figure 10:**
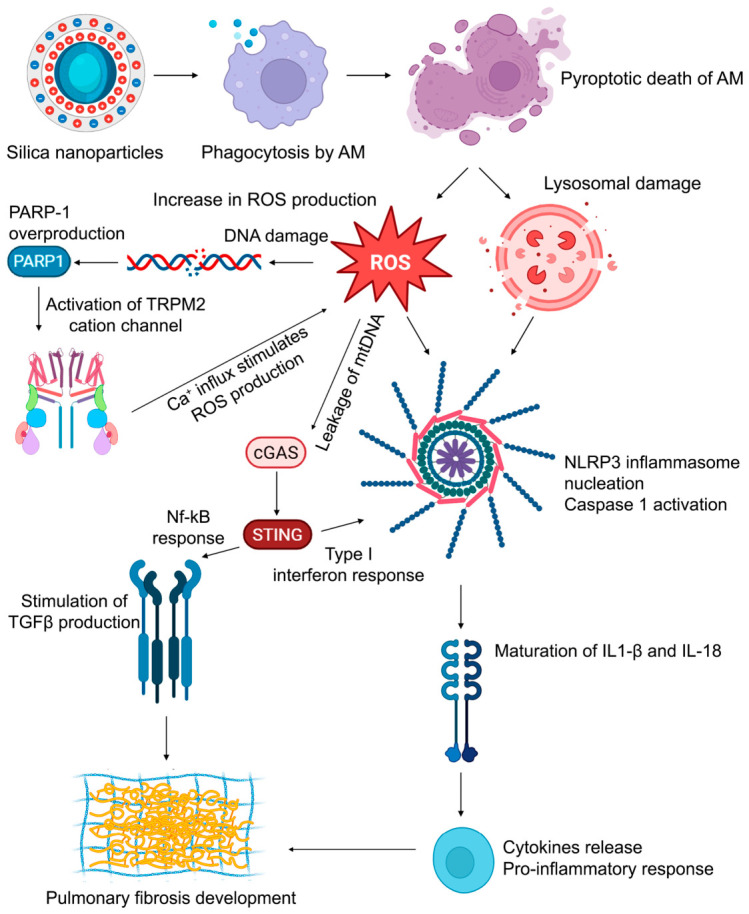
Key molecular pathways involved in the development of silica-induced pulmonary fibrosis (created in BioRender). The phagocytosis of SiNPs by alveolar macrophages (AMs) triggers simultaneously lysosomal damage and a burst of mitochondrial ROS, which together activate the NLRP3 inflammasome, cyclic GMP-AMP synthase (cGAS)/Stimulator in Interferon Genes (STING), and the ROS/PARP/ transient receptor potential melastatin 2 (TRPM2) signaling pathways perpetuating pro-inflammatory and pro-fibrotic processes.

At the molecular level, responses to SiNPs are conditioned by the NLRP3 inflammasome, cyclic GMP-AMP synthase (cGAS)/Stimulator in Interferon Genes (STING), and the ROS/PARP/ transient receptor potential melastatin 2 (TRPM2) signaling pathways (Figure 10). The phagocytosis of SiNPs by alveolar macrophages (AMs) serves as the critical initiating event, simultaneously triggering lysosomal damage and a burst of mitochondrial ROS, which together activate these three pathways.

The central role of the NLRP3 inflammasome in SiNP-induced inflammation is well-established. Phagolysosome rupture releases cathepsin B into the cytosol that, together with K^+^ efflux, promotes NLRP3 nucleation. This leads to caspase-1 activation and the maturation of pro-inflammatory cytokines IL-1β and IL-18. [56]. At the same time, SiNP-induced ROS production leads to oxidative stress and causes significant DNA damage, leading to hyperactivation of PARP-1 [57]. Consequent depletion of nicotinamide adenine dinucleotide (NAD^+^) and accumulation of ADP-ribose (ADPR) polymers directly activate the TRPM2 cation channel [58]. The resulting TRPM2-mediated Ca^2+^ influx serves as a critical second messenger, amplifying mitochondrial damage and directly facilitating NLRP3 inflammasome assembly [59].

Simultaneously, the cGAS/STING pathway is a parallel responder to the SiNP-induced cellular stress. The oxidative mitochondrial damage that feeds into the PARP/TRPM2 axis also compromises mitochondrial membrane integrity, leading to the leakage of oxidized mitochondrial DNA (mtDNA) into the cytosol [60]. This mtDNA is a potent ligand for cGAS, to activate STING on the endoplasmic reticulum. STING activation proceeds via both canonical (IRF3/type I interferon) and non-canonical (NF-κB) arms, perpetuating a pro-inflammatory and pro-fibrotic transcriptional program. The STING-induced type I interferon response can prime NLRP3 expression, while the TRPM2-mediated calcium influx can sensitize cGAS/STING signaling by promoting mitochondrial stress [61]. This establishes a feed-forward loop wherein initial SiNP-mediated damage activates the rapid, ROS/PARP/TRPM2/NLRP3-dependent arm and the delayed, sustained cGAS/STING-dependent arm of the inflammatory response.

The self-amplifying loop of these pathways transitions acute inflammation to chronic fibrosis. STING signaling in both macrophages and damaged epithelial cells directly promotes TGF-β production and amplifies TGF-β receptor signaling, a master regulator of collagen deposition. Therefore, the fibrotic outcome is the integrated result of the following: (i) ROS/PARP/TRPM2 provides the immediate, Ca^2+^-, and NAD^+^-mediated signal amplification; (ii) NLRP3 translates this into a cascade of inflammasome-dependent cytokines; (iii) cGAS/STING sustains the response and activates TGF-β machinery. This mechanistic framework elucidates the intertwined molecular pathogenesis of SiNP-induced lung disease (Figure 10).

In conclusion, by combining clinical phenotyping with an animal model, this study may establish a translational framework for SiNP-induced lung remodeling and fibrosis, one of the main features of O-COPD. We confirm silica nanoparticles as a key driver of a fibrotic O-COPD phenotype and present an actual model that to a certain extent captures the essence of its chronic, progressive, and irreversible nature.

## 5. Limitations of the Study

This study has several limitations. First is the low number of mice per group, which is acceptable for initial study, but additional studies, using larger number of animals, are necessary to confirm the findings obtained in the present study. Second is the possible error in the detection of Si accumulation in the lung and lymph node tissues due to the individual characteristics of the organism, such as different effective fractions of nanoparticles that reach the lungs for the former as well as different clearance speed of nanoparticles from lung tissue by the lymphatic system for the latter. Third is the fact that murine model of SiNP-induced lung inflammation and fibrosis does not fully recapitulate key clinical and morphological features of human O-COPD, which limits the usefulness of this model for direct comparison with clinical data. Fourth limitation is the lack of lung function tests in murine models, limiting its potential use as a platform for nanoparticle effect study unless further studies are conducted to elucidate the functional parameters of the lungs in this model. Fifth is the lack of the assessment of dose–response effect in the lungs upon nanoparticle administration. In order to obtain clinically significant data that can be used to modify the safety regulations at the workplace, future studies should assess the No Observed Adverse Effect Levels (NOAELs) of SiNPs. Finally, more human data are needed for comparison with the future animal studies proposed above.

## 6. Conclusions

The present study shows that the phenotypes of O-COPD, chronic lung disorder attributable to occupational exposures, are associated with the chemical composition of industrial aerosols. Our data also indicate that fibrotic changes are the predominant alterations in the lungs of workers exposed to silica nanoparticles (SiNPs). Data obtained in our murine model successfully replicated the key characteristics of human O-COPD, including silicotic nodule formation and progressive airway remodeling/fibrosis. Since these data agreed with the O-COPD patient cohort, they indicate that future studies using mouse model based on the one in the present study are a feasible means of obtaining data that can be used for assessment of SiNP inhalation toxicity and the setting of workplace safety regulations.

## Figures and Tables

**Table 1:** Associations between the mass concentration and chemical composition of nanoparticles in industrial aerosols and O-COPD phenotypes.

Predictor	Predicted Parameter	B	R	R^2^	*p*-Value
Mass concentration of metal nanoparticles, µg/mL	DLco, %	−1.2	0.85	0.80	0.002
	FEV1, %	−0.8	0.75	0.70	0.015
	Eosinophilic inflammation	1.2	0.92	0.89	0.001
	Low attenuation area (LAA, %)	1.2	0.88	0.77	0.002
Mass concentration of silica nanoparticles, µg/mL	DLco, %	−1.6	0.90	0.87	0.001
	FEV1, %	−0.8	0.85	0.79	0.005
	Paucigranulocytic inflammation	1.4	0.92	0.88	0.001
	High attenuation area (HAA, %)	2.2	0.91	0.83	0.001

## Data Availability

The data in this article will be made available upon reasonable request to the corresponding author.

## Supplementary Materials

The following supporting information can be downloaded at https://www.mdpi.com/article/10.3390/nano16140866/s1. Figure S1: Characteristics of silica nanoparticles (SiNPs) used in the study; Figure S2: Characteristics of magnetic nanoparticles (MNPs) used in the study; Figure S3: Gating strategy of the flow cytometric characterization of peripheral blood immune cells of experimental animals; Figure S4: Histological structure of lymph nodes after SiNP administration; Figure S5: Blood immune cells profile after SiNP administration. Table S1: Inclusion, non-inclusion and exclusion criteria for patients in the study; Table S2: Primary and phenotypic characteristics of patients included in the study; Table S3: Primary and phenotypic characteristics of O-COPD patients depending on the chemical composition of industrial aerosols.

## Abbreviations

The following abbreviations are used in this manuscript:

O-COPD	Occupational chronic obstructive pulmonary disease
SiNPs	Silica nanoparticles
MNPs	Magnetic nanoparticles
CAT	COPD assessment test
FEV1	Forced expiratory volume in 1 s
FVC	Forced vital capacity
DLco	Lung diffusion capacity for carbon monoxide
CT	Computed tomography
TEOS	Tetraethoxysilane
RMST	Restricted mean survival time
ECM	Extracellular matrix
ICP-AES	Inductively coupled plasma atomic emission microscopy
LAA	Low attenuation area
HAA	High attenuation area
mPAP	Mean pulmonary artery pressure
AMs	Alveolar macrophages
AECs	Alveolar epithelial cells
