# Supplementary material for: Deciphering Pathogenesis of Silica Nanoparticle-Induced Airway Remodeling and Fibrosis: Insights from a Human Patient Cohort and a Murine Model
Source: Nanomaterials (Basel). 2026 Jul 15;16(14):866. doi: 10.3390/nano16140866 (PMC13414849; doi:10.3390/nano16140866)
Supplement: Supplementary file 1 [file nanomaterials-16-00866-s001.zip › nanomaterials-4413422-supplementary.pdf]

Supplementary materials

Table S1. Inclusion, non-inclusion and exclusion criteria for patients in the study.

**Inclusion Criteria**
- Informed consent to participate in the study.<br>- A negative PCR result for SARS-CoV-2 RNA from naso- and oropharyngeal swabs and a negative ELISA result for IgM and IgG to SARS-CoV-2 spike proteins at the time of enrollment.<br>- Men and women aged 40 to 65 years.<br>*For the Occupational COPD group:*<br>- A diagnosis of COPD meeting the GOLD 2011 criteria (post-bronchodilator ratio of forced expiratory volume in 1 second [FEV1] to forced vital capacity [FVC] less than or equal to 0.7).<br>- Documented exposure to inorganic dust and/or toxic gases (vapors) at the workplace with levels exceeding the Occupational Exposure Limits (OEL) by 3 times or more, and the presence of nanoparticles in the industrial aerosol due to the nature of technological processes, confirmed by chemical-hygienic studies.<br>- Work experience under the conditions for at least 10 years.<br>- Onset of COPD symptoms after at least 5 years of work under the conditions.<br>*For the Healthy Workers group:*<br>- Absence of detectable diseases or pathological conditions of the bronchopulmonary system identified by standard methods.<br>- Documented exposure to inorganic dust and/or toxic gases (vapors) at the workplace with levels exceeding the OEL by 3 times or more, and the presence of nanoparticles in the industrial aerosol due to the nature of technological processes, confirmed by chemical-hygienic studies.<br>- Work experience under the conditions for at least 10 years.<br>*For the Tobacco-Smoking COPD group:*<br>- A diagnosis of COPD meeting the GOLD 2011 criteria.<br>- No exposure to industrial aerosols or allergens, including those within the MAC limits.<br>- Tobacco smoking for at least 10 years.<br>- Pack-year index of 10 or more.
**Non-inclusion criteria**
- Absence of informed consent from the patient to participate in the study.<br>- Other diseases of the bronchopulmonary system (simple bronchitis and bronchial asthma were not considered reason for non-inclusion).<br>- History of lung resection or lung volume reduction surgery.<br>- Diseases and conditions other than COPD accompanied by eosinophilia.<br>- Inflammatory diseases other than COPD, including autoimmune and infectious diseases.<br>- Malignant neoplasms, regardless of location.<br>- Left ventricular chronic heart failure stages IIA, IIB, III.<br>- Chronic kidney disease stage C5.<br>- Liver cirrhosis class B-C according to Child-Pugh.<br>- Presence of contraindications to the diagnostic procedures outlined in the study protocol.
**Exclusion criteria**
- Patient's decision to discontinue participation in the study.<br>- Development of other chronic diseases of the bronchopulmonary system besides COPD (simple bronchitis and bronchial asthma were not considered reason for non-inclusion).<br>- Lung resection or lung volume reduction surgery performed during the study.<br>- Development of diseases and conditions other than COPD accompanied by eosinophilia.<br>- Development of other inflammatory diseases besides COPD, including autoimmune and infectious diseases.<br>- Identification of contraindications to the diagnostic procedures outlined in the study protocol.

Table S2. Primary and phenotypic characteristics of patients included in the study.

Parameter	Workers, exposed to industrial aerosols		Tobacco-smoking COPD n=50	Healthy volunteers (control) n=50	p-value
	O-COPD n=50	Apparently healthy n=50			
Men, n (%)<br>Women, n (%)	47 (94%)<br>3 (6%)	46 (92%)<br>4 (8%)	46 (92%)<br>4 (8%)	46 (92%)<br>4 (8%)	0.25
Age, years (Me, IQR)	58<br>(54; 63)	57<br>(54; 61.5)	60<br>(55; 62)	57<br>(54; 59)	0.31
Smokers, n (%)	16 (32%)	17 (34%)	50 (100%)	17 (34%)	0.001
Pack-years index (Me, IQR)	15 (11; 19)	14 (10; 18)	17 (13; 19)	17 (12; 19)	0.15
Duration of smoking, years (Me, IQR)	28 (24; 30)	25 (21; 28)	25 (21; 26)	24 (20; 26)	0.45
Length of occupational exposure, years (Me, IQR)	23 (19; 26)	21 (18; 26)	N/A	N/A	0.25
COPD duration, years (Me, IQR)	12 (8; 15)	N/A	14 (10; 16)	N/A	0.52
Smoking history at the time of COPD symptoms emergence, years (Me, IQR)	15 (13; 16)	N/A	17 (16; 20)	N/A	0.008
Forced expiratory volume in 1 second, FEV1, %	56 (50; 62)	102<br>(99; 105)	51 (49; 61)	101<br>(95; 102)	0.01
Changes in FEV1 after bronchodilator, %	8.2±3.4	N/A	5.9±4.2	N/A	0.003
Forced vital capacity, FVC, %	71 (68; 82)	104<br>(102; 110)	93 (90; 96)	98 (93; 102)	0.01
Diffusion capacity for carbon monoxide, DLco, %	43 (37; 48)	91 (85; 94)	75 (71; 78)	N/A	0.01
Low attenuation area, LAA, %	24.5<br>(22.1; 26.3)	8 (7; 10)	28.5<br>(25.2; 31.9)	N/A	0.15
High attenuation area, HAA, %	45.2<br>(43.1; 47.8)	10 (9; 12)	10.5<br>(7.3; 12.9)	N/A	0.001
Mean pulmonary artery pressure, mPAP, mm Hg	41.3<br>(30.2; 48.5)	17 (11; 19)	17.6<br>(15.1; 30.9)	N/A	0.01
Eosinophilic inflammation, n (%)	11 (22%)	N/A	15 (31.3%)	N/A	0.01
Neutrophilic inflammation, n (%)	7 (14%)	N/A	18 (37.5%)	N/A	0.01
Paucigranulocytic inflammation, n (%)	29 (58%)	N/A	2 (4.2%)	N/A	0.01
Six-minute walking distance, m	352<br>(341; 363)	710<br>(603; 932)	315<br>(303; 317)	N/A	0.42

COPD Assessment Test (CAT) symptom score, points	8 (6; 12)	N/A	9.5 (7; 12)	N/A	0.45

Significance of differences was assessed relative to the tobacco-smoking COPD group. N/A – not applicable.

Table S3. Primary and phenotypic characteristics of O-COPD patients depending on the chemical composition of industrial aerosols.

Parameter	O-COPD n=50		Tobacco-smoking COPD n=50	Healthy volunteers (control) n=50	p-value
	Exposure to metal nanoparticles n=26	Exposure to silica nanoparticles n=24			
Men, n (%)<br>Women, n (%)	24 (92.3%)<br>2 (7.7%)	23 (95.8%)<br>1 (4.2%)	46 (92%)<br>4 (8%)	46 (92%)<br>4 (8%)	0.458<br>0.458
Age, years (Me, IQR)	57 (55; 62)	58 (54; 63)	60 (55; 62)	57 (54; 59)	0.322
Smokers, n (%)	8 (30.8%)	8 (33.3%)	50 (100.0%)	17 (34.0%)	0.001
Pack-year index (Me, IQR)	15 (11; 18)	14 (12; 15)	17 (13; 19)	17 (12; 19)	1.155
Smoking duration, years (Me, IQR)	24 (20; 26)	23 (21; 24)	25 (21; 26)	24 (20; 26)	0.239
Occupational exposure duration, years (Me, IQR)	22 (19; 25)	21 (20; 25)	N/A	N/A	0.316
COPD duration, years (Me, IQR)	12 (8; 15)	13 (9; 15)	14 (10; 16)	N/A	0.52
Occupational exposure duration at onset of chronic respiratory symptoms, years (Me, IQR)	11 (10; 14.5)	10 (10; 13)	N/A	N/A	0.208
Forced expiratory volume in 1 second, FEV1, %	38 (35; 42)	58 (55; 61)	50 (45; 56)	101 (95; 102)	0.002[1,2,3]
Change in FEV1 after bronchodilator, %	10 (9; 10)	4 (3; 6)	5 (4; 6)	N/A	0.001[1,2,3]
Forced vital capacity, FVC, %	57 (55; 64)	83 (79; 88)	81 (78; 84)	98 (93; 102)	0.001[1,2]
Diffusing capacity for carbon monoxide per unit of alveolar volume, DLco/Va, %	34 (31; 38)	48 (42; 54)	55 (52; 65)	N/A	0.001[1,2,3]
Mean	35 (29; 38)	24 (22; 26)	19 (17; 23)	N/A	0.005[1,2,3]

pulmonary artery pressure, mPAP, mm Hg					
Eosinophilic inflammation, n (%)	14 (53.8)	5 (20.8)	17 (34)	N/A	0.048[1,2,3]
Neutrophilic inflammation, n (%)	5 (19.2)	7 (29.2)	26 (52)	N/A	0.012[1,2,3]
Paucigranulocytic inflammation, n (%)	6 (23.1)	11 (45.8)	5 (10)	N/A	0.002[1,2,3]

Significance of differences: 1 – between two O-COPD groups; 2 – between O-COPD with metal nanoparticle exposure and tobacco-smoking COPD; 3 – between O-COPD with silica nanoparticle exposure and tobacco-smoking COPD. N/A – non applicable.

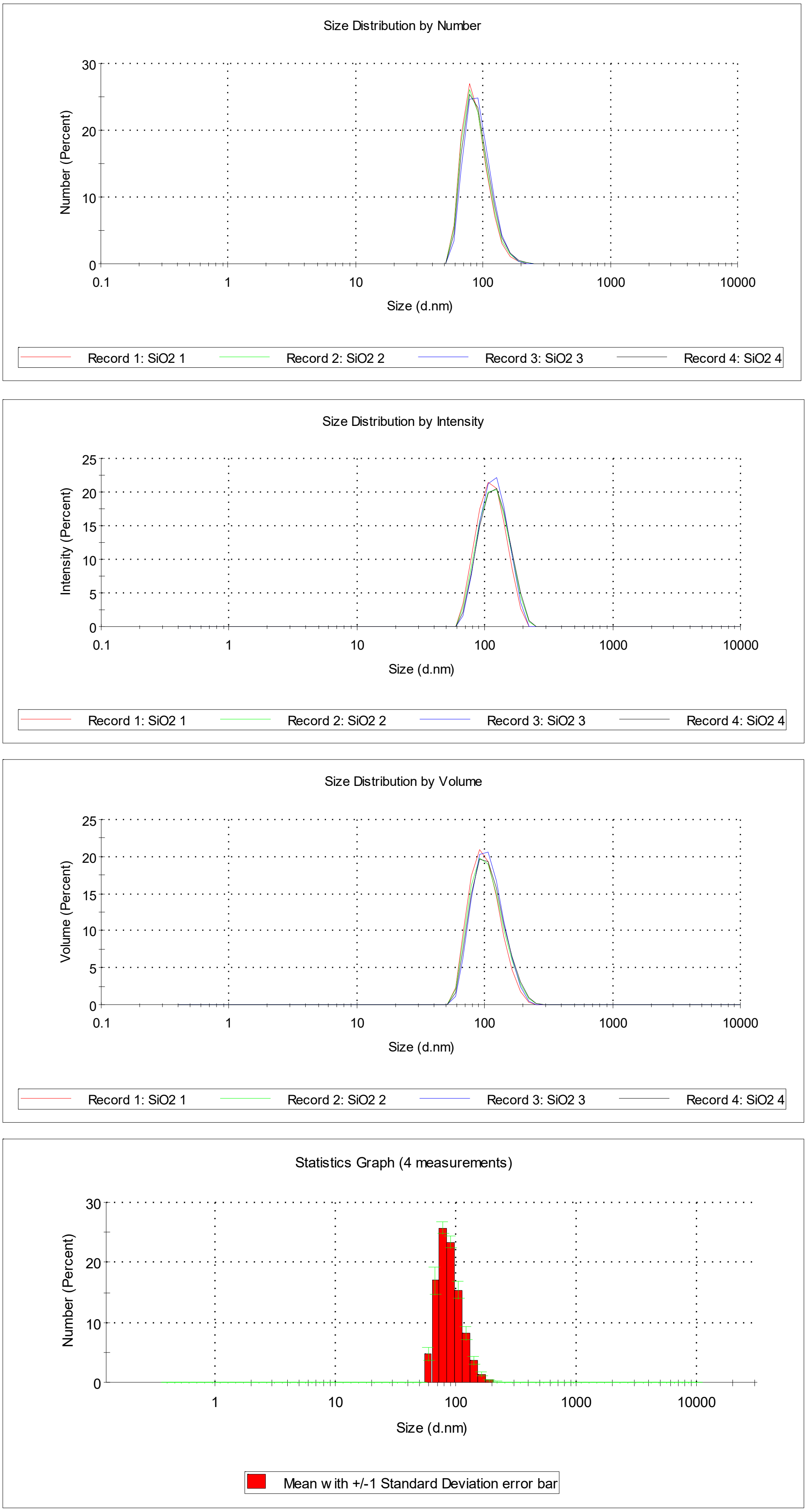

Figure S1. Characteristics of silica nanoparticles (SiNPs) used in the study.

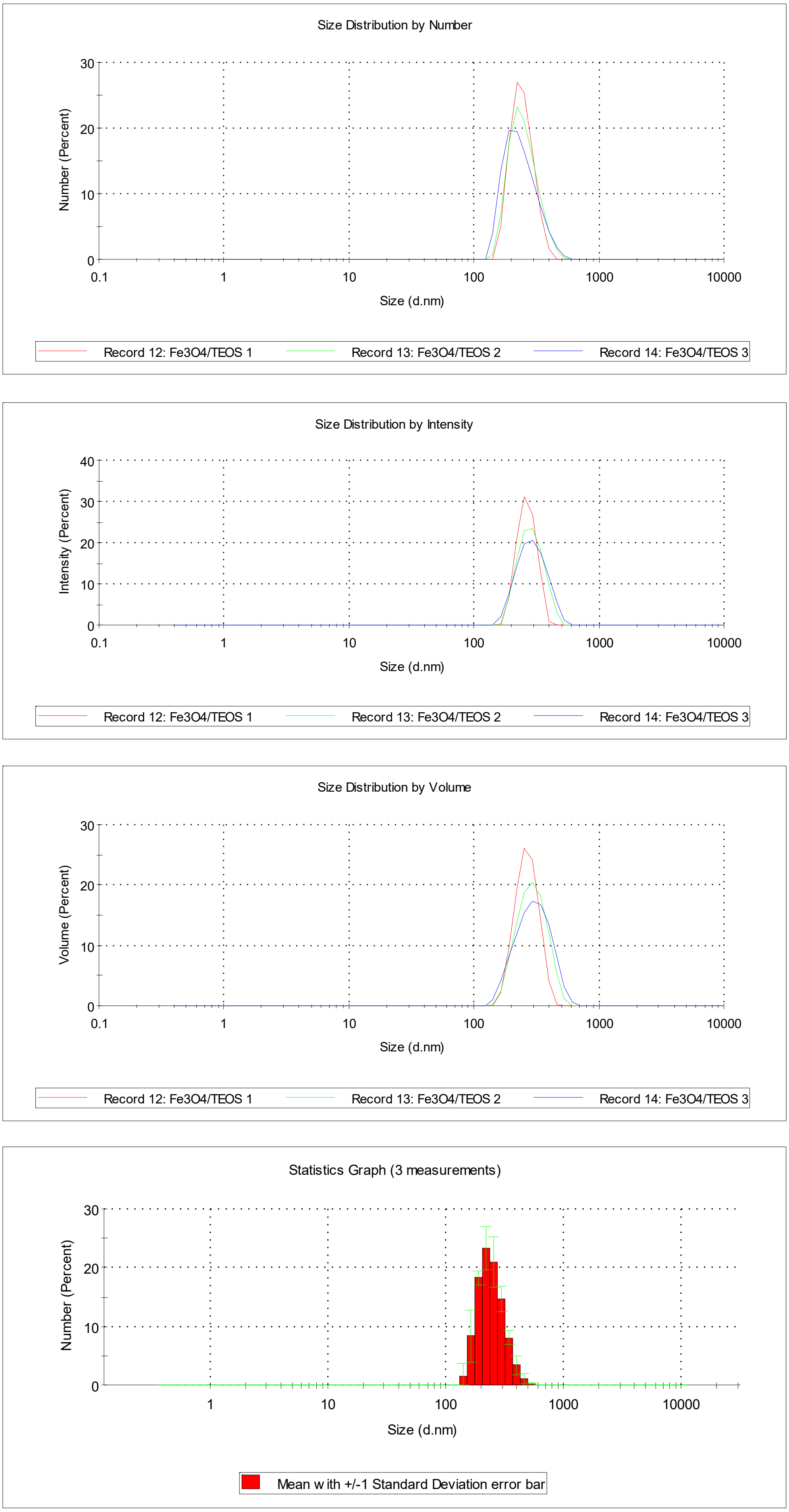

Figure S2. Characteristics of magnetic nanoparticles (MNPs) used in the study.

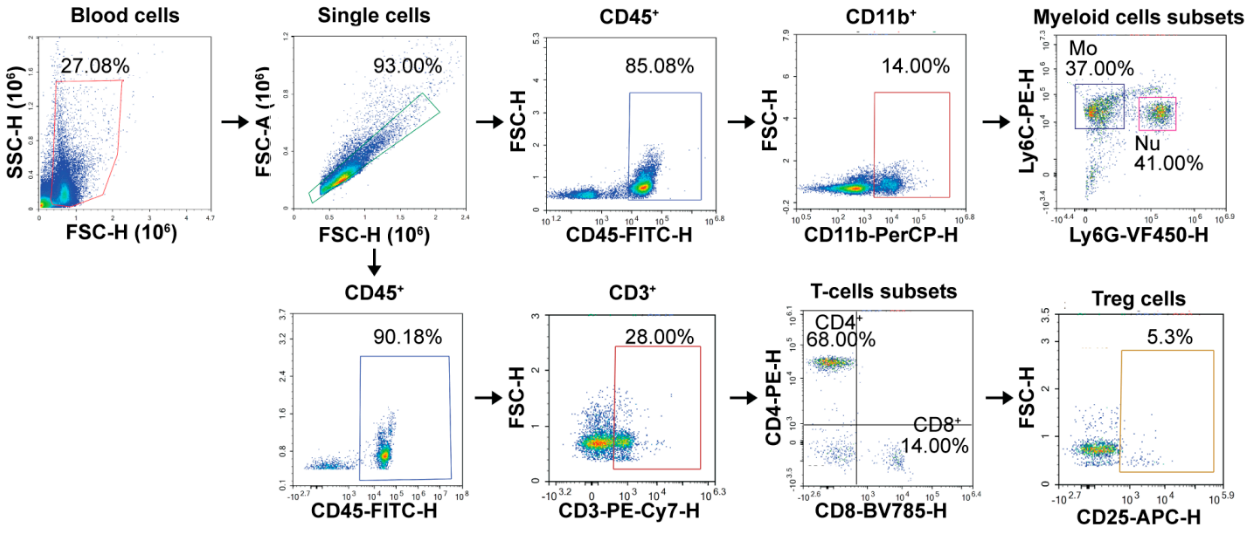

Figure S3. Gating strategy for the flow cytometric characterization of peripheral blood immune cells of experimental animals. Mo – monocytes, Nu – neutrophils, $CD4^+$ – T helper cells, $CD8^+$ – cytotoxic T cells.

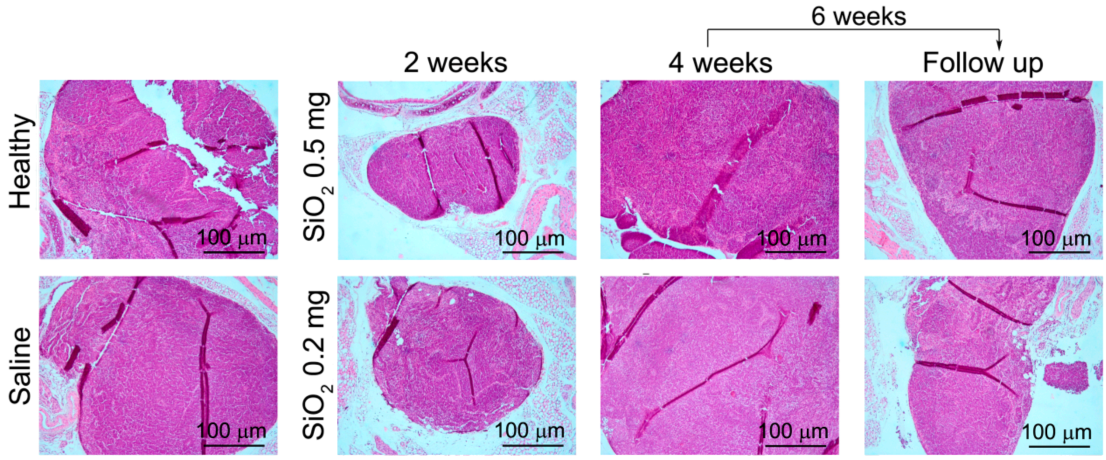

Figure S4. Histological structure of lymph nodes after SiNPs administration. Hematoxylin and eosin staining. Original magnification ×200.

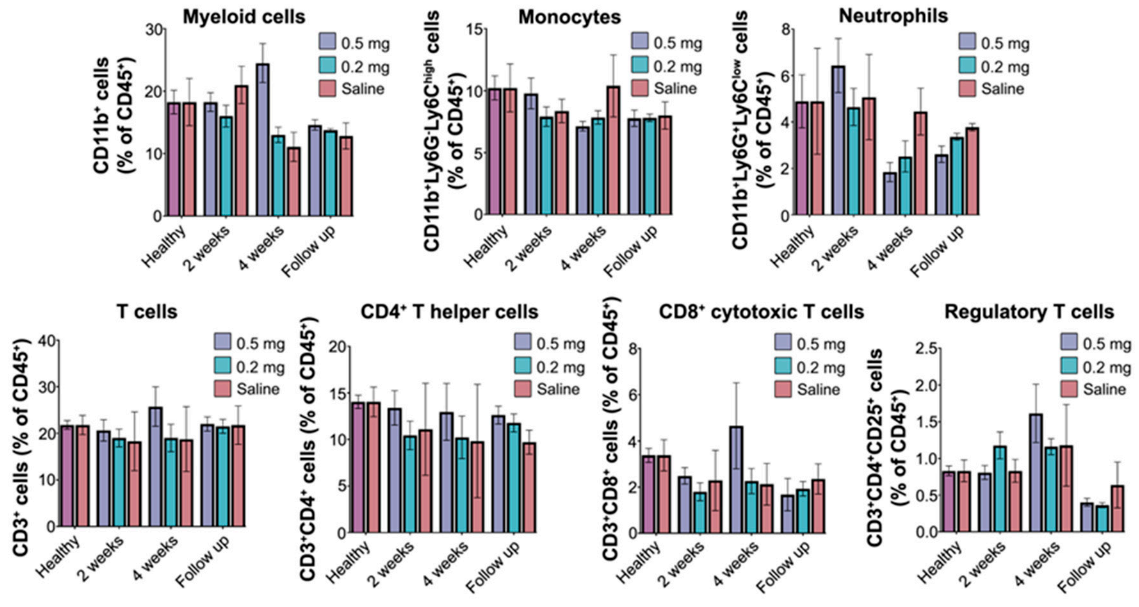

Figure S5. Blood immune cells profile after SiNPs administration. Distribution of $CD11b^+$ cells, monocytes, neutrophils (upper panel), total T cells, $CD4^+$ T cells, $CD8^+$ T cells, and regulatory T cells (bottom panel) in peripheral blood of healthy and SiNPs administered mice.
